# Prodh2‐Mediated Mitochondrial Stress Drives TNF‐α‐Induced Myoblast Dysfunction and Sarcopenia in COPD

**DOI:** 10.1111/acel.70739

**Published:** 2026-10-01

**Authors:** Gang Chen, Zhitao Shangguan, Jingjing Gong, Dalu Cheng, Penghong Chen, Jiandong Li, Qiong Lin

**Affiliations:** ^1^ Department of Orthopedics Fujian Medical University Union Hospital Fuzhou Fujian China; ^2^ Department of Cardiology The First Affiliated Hospital of Fujian Medical University Fuzhou Fujian China; ^3^ Department of Clinical Medicine Fujian Medical University Fuzhou Fujian China; ^4^ Department of Plastic Surgery The Second Affiliated Hospital of Fujian Medical University Fuzhou Fujian China; ^5^ Department of Respiratory and Critical Care Medicine Fujian Medical University Union Hospital Fuzhou Fujian China

**Keywords:** cGAS/STING, chronic obstructive pulmonary disease, mitochondrial DNA, Prodh2, sarcopenia

## Abstract

Sarcopenia in chronic obstructive pulmonary disease (COPD) is driven by elevated tumor necrosis factor‐alpha (TNF‐α), yet the underlying mechanism remains unclear. Here, we identify the mitochondrial metabolic enzyme Prodh2 as a pivotal mediator. Prodh2 was significantly upregulated in sarcopenic COPD patients and a corresponding mouse model. Mechanistically, TNF‐α induced Prodh2 transcription in myoblasts via a p53‐dependent pathway. This upregulation triggered excessive mitochondrial reactive oxygen species, leading to the release of mitochondrial DNA (mtDNA). Cytosolic mtDNA subsequently activated the cGAS/STING innate immune pathway, causing myoblast apoptosis, proliferation arrest, and atrophy. While Prodh2 knockdown reversed these effects in vitro, muscle‐specific knockdown of Prodh2 in vivo critically ameliorated muscle atrophy and restored strength. Our study unveils a novel TNF‐α‐p53‐Prodh2‐cGAS/STING axis that links chronic inflammation to sarcopenia through metabolic‐immune crosstalk, validating Prodh2 as a promising therapeutic target.

## Graphical abstract

TNF‐α upregulates Prodh2 via p53 in COPD myoblasts, driving mitochondrial ROS–induced cGAS/STING activation and myoblast dysfunction. This metabolic‐immune pathway underlies sarcopenia, positioning Prodh2 as a therapeutic target for COPD‐associated muscle wasting.

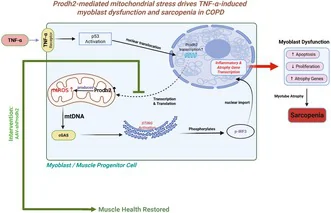

## 1 Introduction

Chronic obstructive pulmonary disease (COPD) is a common respiratory disease characterized by persistent airflow limitation and a chronic inflammatory response, and it has become a leading cause of disability and mortality worldwide (Agedew et al. 2021). The pathological impact of COPD extends far beyond the lungs and is often accompanied by multiple extrapulmonary comorbidities (Park et al. 2022; Solidoro et al. 2022). Among these, sarcopenia—the progressive loss of skeletal muscle mass, strength, and function—is one of the most common and devastating complications. Its prevalence is as high as 32%–33% in COPD patients and is closely associated with reduced exercise capacity, worsened quality of life, and an increased risk of mortality (Nan et al. 2023; Jiang et al. 2023). Systemic chronic inflammation is considered a key pathophysiological link connecting COPD with sarcopenia (Sepulveda‐Loyola et al. 2024). In this context, the key pro‐inflammatory cytokine, tumor necrosis factor‐alpha (TNF‐α), has become a focus of research. Abundant evidence indicates that both circulating and local skeletal muscle levels of TNF‐α are significantly elevated in COPD patients with sarcopenia and are positively correlated with the severity of muscle atrophy (Byun et al. 2017). Skeletal muscle homeostasis and post‐injury repair are highly dependent on the normal function of muscle stem cells and their progenitors. However, although the clinical association between TNF‐α and muscle atrophy is well‐established, the underlying molecular signaling pathways through which it directly impairs muscle cells, including mature myofibers and myogenic progenitors, leading to their dysfunction and ultimately driving sarcopenia, remain largely unclear. Therefore, exploring these mechanisms to identify novel therapeutic targets holds great clinical significance.

Skeletal muscle function is highly dependent on mitochondria, and mitochondrial dysfunction is now recognized as a core pathophysiological feature in the development and progression of sarcopenia (Kumar et al. 2021; Wang, du, et al. 2024). Central to this issue is the integrity of mitochondrial DNA (mtDNA). Compelling evidence has established a causal relationship between the accumulation of mtDNA mutations and the induction of skeletal muscle apoptosis and sarcopenia (Hiona et al. 2010). Under conditions of severe cellular stress, damaged mitochondria can release mtDNA fragments into the cytoplasm, where these mislocalized mtDNA molecules act as critical damage‐associated molecular patterns (DAMPs; Picca et al. 2018). Pro‐inflammatory cytokines, particularly TNF‐α, have been identified as key upstream triggers driving this pathological process. Indeed, studies have confirmed that TNF‐α directly impairs mitochondrial function and leads to destructive mtDNA damage, a phenomenon notably evident in COPD and other muscle‐wasting disorders (Luo et al. 2024; Remels et al. 2010).

Proline dehydrogenase 2 (Prodh2), as a molecule of significant interest, is a metabolic enzyme localized within the mitochondria. Crucially, its catalytic reaction, the degradation of hydroxyproline, is an established and direct source of mitochondrial reactive oxygen species (mtROS; Kazberuk et al. 2022). Furthermore, the *Prodh2* gene has been explicitly identified as a direct transcriptional target of the pivotal stress‐response protein, p53, with its expression being activated by p53 (Cooper et al. 2008). These established characteristics place Prodh2 at a critical nexus of cellular metabolism, oxidative stress, and signal transduction. However, while its functions have been progressively studied in fields such as oncology (Gong, Li, et al. 2025; Tang et al. 2018), the role of Prodh2 in skeletal muscle pathophysiology, particularly in the development of sarcopenia, remains a completely unexplored area. This reveals a significant knowledge gap and posits that Prodh2 may act as a critical molecular switch that translates chronic inflammatory signals into mitochondrial stress and subsequent myoblast dysfunction in sarcopenia.

Therefore, this study aimed to elucidate the core molecular mechanisms of COPD‐related sarcopenia and to identify novel intervention targets. In this research, we combined analyzes of clinical samples, the establishment of an animal model, and experiments with C2C12 cells. A key molecule was identified through high‐throughput sequencing, and its role in mediating a complete signaling pathway from mitochondrial stress to downstream immune activation was systematically dissected through a series of in vitro and in vivo functional experiments. Our findings not only provide a novel perspective on the pathogenesis of this disease but also offer a crucial theoretical basis and a potential target for the development of targeted therapeutic strategies.

## 2 Material and Methods

### 2.1 Clinical Sample Collection and Processing

This study was conducted in accordance with the principles of the Declaration of Helsinki and was approved by the Institutional Review Board of Fujian Medical University Union Hospital (Approval No: 2023KJCX026). Written informed consent was obtained from all participants prior to sample collection. A total of three groups of subjects were enrolled in this study: healthy controls (HC, *n* = 10), patients with chronic obstructive pulmonary disease without sarcopenia (COPD, *n* = 10), and patients with COPD and sarcopenia (COPD+Sarcopenia, *n* = 10). The diagnosis of COPD and the assessment of sarcopenia were performed according to the Global Initiative for Chronic Obstructive Lung Disease (GOLD) guidelines and the latest diagnostic criteria from the Asian Working Group for Sarcopenia (AWGS), respectively. Fasting venous blood samples (5 mL) were collected from all subjects in the morning. After collection, blood samples were allowed to clot at room temperature for 30 min and then centrifuged at 3000 rpm for 15 min at 4°C. The resulting supernatant (serum) was carefully collected, aliquoted into RNase‐free cryotubes, and immediately stored at −80°C until further analysis.

### 2.2 Cell Culture and Treatment

The mouse C2C12 myoblast cell line was purchased from BeNa Culture Collection. Cells were cultured in high‐glucose Dulbecco's Modified Eagle's Medium (DMEM, C0891; Beyotime) supplemented with 10% Fetal Bovine Serum (FBS, C0226S; Beyotime) and 1% Penicillin–Streptomycin solution (C0222; Beyotime). Cells were maintained in a humidified incubator at 37°C with 5% CO_2_. To establish an in vitro model of cellular dysfunction, C2C12 cells in the logarithmic growth phase were seeded into culture plates. After cell attachment, the medium was replaced with fresh medium containing a concentration gradient (0, 5, 10, 20 ng/mL) of recombinant mouse TNF‐α (P5318‐10; Beyotime). The cells were then incubated for 24–72 h, depending on the specific experimental requirements.

### 2.3 Animals and Model Establishment

SPF male C57BL/6J mice (8–10 weeks old, 20–25 g) were obtained from the Animal Center of Fujian Medical University and housed in an SPF facility under controlled conditions of temperature (22°C ± 2°C), humidity (50% ± 10%), and a 12‐h light/dark cycle, with ad libitum access to food and water. All animal experimental protocols were approved by the Institutional Animal Care and Use Committee of Fujian Medical University (Approval No: IACUC FJMU 2024‐Y‐1872).

Mice were randomly assigned to the following groups. Sham group: housed in a normal air environment for 24 weeks. COPD model group: subjected to chronic cigarette smoke (CS) exposure for 24 weeks. COPD with sarcopenia model group (Se‐Copd): subjected to 24 weeks of CS exposure combined with hindlimb unloading (HU) for the final 2 weeks to induce muscle atrophy.

For therapeutic intervention experiments, Se‐Copd model mice were further randomized into the following groups: the Model + AAV‐shNC group and the Model + AAV‐shProdh2 group received intramuscular multi‐point injections of the corresponding shRNA‐carrying AAV into the gastrocnemius muscle at a dose of 1 × 10^11^ viral genomes per mouse. The AAV was administered as a single injection at the beginning of the intervention period (Week 1). The Model + Anti‐TNF‐α group received intraperitoneal injections of a TNF‐α neutralizing antibody (Proteintech, 69,002‐1‐Ig) at a dose of 10 mg/kg twice a week for the entire 12‐week intervention period (totaling 24 injections), and the Combination Therapy group received both intramuscular AAV‐shProdh2 and intraperitoneal Anti‐TNF‐α. The intervention lasted for 12 weeks. At the end of the experiment, all mice were euthanized under isoflurane anesthesia. Body weight was recorded, and gastrocnemius muscles were rapidly collected and weighed for subsequent analysis.

### 2.4 RNA Extraction, Library Preparation, and Sequencing

C2C12 cells in the logarithmic growth phase were divided into a control group and a TNF‐α‐treated group (*n* = 3 per group). After treatment, total RNA was extracted from the cells using TRIzol reagent (R0016; Beyotime). The concentration and integrity of the RNA were assessed using a NanoDrop spectrophotometer and an Agilent 2100 Bioanalyzer. Qualified RNA samples were sent to Sangon Biotech (Shanghai) Co. Ltd. for library construction and sequencing. Briefly, mRNA was enriched from total RNA using oligo(dT)‐attached magnetic beads and then fragmented. The fragmented mRNA served as a template for first‐strand cDNA synthesis, followed by second‐strand synthesis and purification. The purified double‐stranded cDNA underwent end‐repair, A‐tailing, and ligation with sequencing adapters. Finally, the cDNA libraries were constructed by PCR amplification. After quality assessment using a Qubit 2.0 fluorometer and an Agilent 2100 system, the libraries were sequenced on an Illumina NovaSeq platform with a paired‐end strategy.

### 2.5 Grip Strength Test

One day before the end of the experiment, forelimb muscle strength of all mice was assessed using a grip strength meter. Before the test, mice were acclimatized to the testing environment for at least 30 min. During the test, the operator held the mouse horizontally by the base of its tail, allowing it to naturally grasp the metal pull bar of the instrument with its forepaws. The mouse was then pulled backward horizontally at a constant speed of approximately 2 cm/s until its grip was released. The instrument automatically recorded the peak force (in gram‐force, gf). Each mouse was tested five times with a 1‐min rest interval between trials. To ensure data stability, the first and last measurements were discarded, and the average of the three stable, consecutive measurements was used for statistical analysis.

### 2.6 H&E Staining and Cross‐Sectional Area (CSA) Analysis

Freshly dissected mouse gastrocnemius muscles were immediately fixed in 4% paraformaldehyde solution at 4°C for over 24 h. The fixed tissue samples underwent standard processing, including dehydration through a graded ethanol series, clearing in xylene, and embedding in paraffin. The paraffin blocks were sectioned into 4 μm‐thick serial slices using a rotary microtome. Sections were mounted on glass slides, baked at 60°C for 1 h, and then deparaffinized in xylene and rehydrated through a graded ethanol series. Subsequently, the sections were stained using a H&E staining kit (G1005; ServiceBio). After staining, the slides were dehydrated again, cleared in xylene, and coverslipped using neutral balsam. Finally, clear cross‐sectional images of muscle fibers were captured under a 200× magnification using a microscope (BX53; Olympus). The captured images were analyzed using ImageJ software (National Institutes of Health, USA) to quantify the average cross‐sectional area (CSA) of the muscle fibers by manually tracing the outlines of at least 100 fibers per image.

### 2.7 TUNEL Assay for Apoptosis

Apoptosis was detected using the Terminal deoxynucleotidyl transferase dUTP nick end labeling (TUNEL) assay. C2C12 cells were seeded on coverslips placed in 24‐well plates and subjected to the respective treatments. After treatment, the culture medium was discarded, and the cells were washed twice with PBS. Cells were fixed with 4% paraformaldehyde for 15 min at room temperature, followed by permeabilization with 0.3% Triton X‐100 in PBS for 5 min. After another wash with PBS, 50 μL of Cy3‐labeled TUNEL reaction mixture, prepared according to the manufacturer's instructions (C1089; Beyotime), was added to each coverslip. The slides were then incubated in a humidified chamber at 37°C for 60 min in the dark. The reaction was terminated by washing with PBS. Finally, the coverslips were counterstained with DAPI and mounted using an anti‐fade mounting medium. Images were captured using a fluorescence microscope (BX53; Olympus), and the apoptosis rate was assessed by calculating the percentage of red‐fluorescent (TUNEL‐positive) nuclei relative to the total number of blue‐fluorescent (DAPI‐stained) nuclei.

### 2.8 EdU (5‐Ethynyl‐2′‐Deoxyuridine) Incorporation Assay for Cell Proliferation

Cell proliferation activity was assessed using an EdU incorporation assay. C2C12 cells were seeded on coverslips in 24‐well plates and subjected to the designated treatments. Two hours before the end of the treatment period, EdU solution (C0075S; Beyotime) was added to the culture medium to a final concentration of 50 μM, and the cells were returned to the incubator. After incubation, cells were fixed with 4% paraformaldehyde and permeabilized with 0.5% Triton X‐100. Subsequently, a Click‐iT reaction cocktail was prepared according to the kit's instructions, added to the coverslips, and incubated for 30 min at room temperature in the dark. After the reaction, cells were washed with PBS and counterstained with Hoechst 33342 (C1022; Beyotime) to visualize the nuclei. Finally, coverslips were mounted with an anti‐fade mounting medium, and images were captured using a fluorescence microscope (BX53; Olympus). The proliferation rate was quantified by calculating the percentage of red‐fluorescent (EdU‐positive) nuclei relative to the total number of blue‐fluorescent (total) nuclei.

### 2.9 LDH Cytotoxicity Assay

Cytotoxicity was evaluated by measuring the release of lactate dehydrogenase (LDH) into the culture medium. C2C12 cells were seeded in 96‐well plates and subjected to the indicated treatments. At the end of the treatment period, the assay was performed according to the instructions of the LDH Cytotoxicity Assay Kit (C0016; Beyotime). Briefly, 120 μL of cell culture supernatant from each well was carefully transferred to a new 96‐well plate. Then, 60 μL of the LDH reaction working solution was added to each well. After gentle mixing, the plate was incubated for 30 min at room temperature, protected from light. Finally, the absorbance at 490 nm was measured using a microplate reader (680; Bio‐Rad). Cytotoxicity was calculated using the formula: (Absorbance_sample − Absorbance_background)/(Absorbance_maximum_enzyme_activity − Absorbance_background) × 100%.

### 2.10 Myotube Differentiation and Morphological Analysis

C2C12 myoblasts were seeded into 12‐well plates at a density of 2 × 10^5^ cells/well. Upon reaching 90%–100% confluency, the growth medium was replaced with differentiation medium (DM), consisting of DMEM supplemented with 2% horse serum (16050122; Gibco) and 1% Penicillin–Streptomycin. The differentiation medium was refreshed every 48 h. After 4 days of differentiation, the resulting myotubes were subjected to the indicated treatments. Following treatment, myotube morphology was captured using an inverted phase‐contrast microscope (BX53; Olympus) equipped with a digital camera. For each well, at least five random, nonoverlapping fields were imaged at 100× magnification. The data parameters of length of the myotubes were measured using ImageJ software.

### 2.11 Enzyme‐Linked Immunosorbent Assay (ELISA)

The concentration of TNF‐α in mouse serum was quantified using a specific ELISA kit (EK0527; Boster) according to the manufacturer's protocol. Briefly, serum samples and a series of serially diluted standards were added to the pre‐coated microplate and incubated at 37°C for 90 min. After washing, a biotinylated detection antibody was added and incubated at 37°C for 60 min. The plate was washed again, followed by the addition of HRP‐conjugated streptavidin and incubation at 37°C for 30 min. After thorough washing, TMB substrate solution was added, and the plate was incubated at 37°C in the dark for 15–20 min for color development. The reaction was terminated by adding a stop solution, and the absorbance (OD) at 450 nm was immediately measured using a microplate reader. The concentration of TNF‐α was calculated based on the standard curve generated from the OD values of the standards.

### 2.12 Quantitative Real‐Time PCR (qRT‐PCR)

Total RNA was extracted from C2C12 cells or mouse gastrocnemius muscle tissue using TRIzol reagent (R0016; Beyotime). One microgram of total RNA was reverse‐transcribed into cDNA using a reverse transcription kit (D7170; Beyotime). Subsequently, qRT‐PCR was performed using SYBR Green Master Mix (D7260; Beyotime) and gene‐specific primers (sequences listed in Table 1) on a Bio‐Rad CFX96 Real‐Time PCR System. The cycling conditions were as follows: an initial denaturation at 95°C for 30 s, followed by 40 cycles of denaturation at 95°C for 5 s and annealing/extension at 60°C for 30 s. Each sample was run in triplicate. The relative expression levels of target genes were calculated using the $2^{-\Delta \Delta C_{t}}$ method, with GAPDH serving as the internal control.

**TABLE 1:** The sequences of all primers.

Gene	Sequence (5′→3′)
Atrogin‐1‐forward	CAGCTTCGTGAGCGACCTC
Atrogin‐1‐reverse	GGCAGTCGAGAAGTCCAGTC
Prodh2‐forward	GCCTTCCATGTCAAGGGAACC
Prodh2‐reverse	CCTGAAACGCTAGTCCATGAGT
MuRF1‐forward	CCAGGCTGCGAATCCCTAC
MuRF1‐reverse	ATTTTCTCGTCTTCGTGTTCCTT
GAPDH‐forward	AGGTCGGTGTGAACGGATTTG
GAPDH‐reverse	GGGGTCGTTGATGGCAACA

For the quantification of cytoplasmic mtDNA, cytosolic fractions were first isolated using a Nuclear and Cytoplasmic Extraction Kit (ab65321; Abcam, Cambridge, MA, USA). DNA was then purified from the cytosolic fractions using a DNA extraction kit (Qiagen, Chatsworth, CA). The relative content of mtDNA was determined by qRT‐PCR, quantifying the mitochondrial‐specific gene *ND1* relative to the nuclear gene *B2M*, which served as the internal control.

### 2.13 Western Blot Analysis

Total proteins were extracted from C2C12 cells or mouse gastrocnemius muscle tissues using RIPA lysis buffer (P0013B; Beyotime) supplemented with protease and phosphatase inhibitors. Protein concentration was determined using a BCA Protein Assay Kit (P0012; Beyotime). Equal amounts of protein (20–40 μg) were separated by 10% or 12% SDS‐PAGE and transferred to polyvinylidene fluoride (PVDF) membranes (IPVH00010; Millipore). The membranes were blocked with 5% non‐fat milk or BSA in TBST for 1 h at room temperature. Subsequently, the membranes were incubated with primary antibodies overnight at 4°C. The primary antibodies used were: Prodh2 (ab203875, 1:1000; Abcam), p‐p53 (Ser15) (BM3987, 1:500; Boster), p53 (ab241566, 1:1000; Abcam), p‐STING (Ser366) (72971S, 1:1000; CST), STING (A01871‐3, 1:1000; Boster), p‐IRF3 (Ser396) (BM4844, 1:500; Boster), IRF3 (A00165‐6, 1:1000; Boster), Cleaved Caspase‐3 (AF1150, 1:500; Beyotime), Atrogin‐1 (A02531‐1, 1:1000; Boster), MuRF1 (ab313852, 1:1000; Abcam), and GAPDH (A00227‐1, 1:2000; Boster) as a loading control. The next day, after washing three times with TBST, the membranes were incubated with HRP‐conjugated secondary antibodies (ab97051, 1:5000; Abcam) for 1 h at room temperature. After further washing, the protein bands were visualized using an ECL kit (P0018S; Beyotime) on a chemiluminescence imaging system. The band intensities were semi‐quantified using ImageJ software.

### 2.14 Mitochondrial ROS Detection

Mitochondrial ROS levels were specifically detected using the MitoSOX Red mitochondrial superoxide indicator (M36008; Thermo Fisher Scientific). C2C12 cells were seeded on coverslips in 24‐well plates and treated as indicated. After treatment, cells were washed once with pre‐warmed PBS and then incubated with serum‐free medium containing 5 μM MitoSOX Red for 10–15 min at 37°C in the dark. Following incubation, cells were gently washed three times with warm PBS and counterstained with DAPI. Coverslips were then mounted with an anti‐fade mounting medium and immediately imaged using a fluorescence microscope.

### 2.15 Mitochondrial Membrane Potential (MMP) Assay

Changes in MMP were assessed using a JC‐1 Assay Kit (C2003S; Beyotime). C2C12 cells were grown on coverslips and subjected to the specified treatments. After treatment, the culture medium was removed, and cells were washed once with PBS. Subsequently, 1 mL of freshly prepared JC‐1 staining solution was added, and the cells were incubated for 20 min at 37°C in the dark. After incubation, the cells were washed twice with JC‐1 staining buffer (1×) to remove excess probe. Images were captured using a fluorescence microscope. At high MMP, JC‐1 forms aggregates in the mitochondrial matrix, emitting red fluorescence. At low MMP, JC‐1 exists as monomers in the cytoplasm, emitting green fluorescence. The level of MMP was evaluated by observing the relative intensity changes between red and green fluorescence.

### 2.16 Statistical Analysis

All quantitative data are presented as the mean ± SD. Statistical analyzes and graphing were performed using GraphPad Prism 9.0 (GraphPad Software, USA). The normality of the data was assessed using the Shapiro–Wilk test. For normally distributed data, comparisons between two groups were performed using an unpaired Student's *t*‐test. For comparisons among multiple groups, an ANOVA was used, followed by either Tukey's post hoc test, depending on the homogeneity of variances. Nonparametric tests were used for data that were not normally distributed. A *p*‐value of < 0.05 was considered statistically significant. Significance levels are denoted as follows: *p < 0.05, **p < 0.01, ***p < 0.001, and ****p < 0.0001. All experiments were performed with at least three independent biological replicates. All quantitative data are presented as the mean ± standard deviation (SD).

## 3 Results

### 3.1 TNF‐α Is Significantly Elevated in Patients and a Mouse Model of COPD With Sarcopenia

To investigate key inflammatory factors in COPD‐related sarcopenia, we first analyzed clinical serum samples. The heatmap results showed that TNF‐α was one of the most significantly upregulated cytokines in patients with COPD and sarcopenia (COPD‐S) (Figure 1A). Subsequently, we established a mouse model of COPD with sarcopenia (Se‐Copd). H&E staining confirmed that, compared to the sham group, Se‐Copd mice exhibited significant pulmonary inflammation and skeletal muscle fiber atrophy (Figure 1B). Furthermore, the muscle fiber size frequency distribution revealed a significant leftward shift in the Se‐Copd group, quantitatively confirming a higher proportion of smaller, atrophic fibers. To validate the changes in key molecules, we measured TNF‐α levels. ELISA results indicated that the serum TNF‐α concentration in the Se‐Copd group was significantly higher than that in the sham group (Figure 1C). Similarly, qPCR and Western blot analyzes revealed that both the mRNA and protein expression levels of Tnf‐α were significantly upregulated in the skeletal muscle tissue of Se‐Copd mice (Figure 1D,E). These findings suggest that TNF‐α plays a critical role in the pathogenesis of COPD with sarcopenia.

**FIGURE 1:**
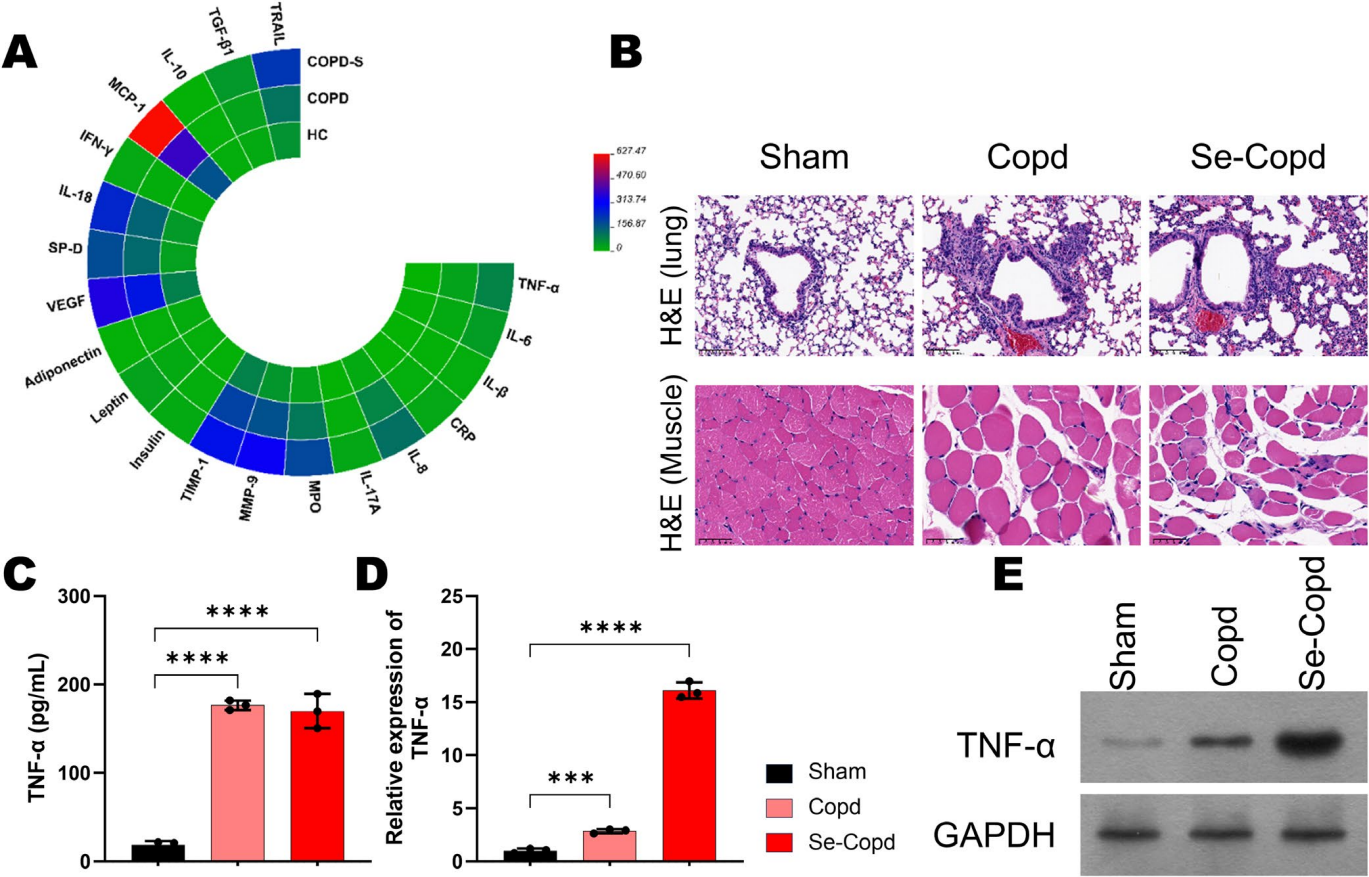
TNF‐α is significantly elevated in patients and a mouse model of COPD with sarcopenia. (A) Circular heatmap illustrating the expression profile of serum cytokines from healthy controls (HC), COPD patients without sarcopenia (COPD), and COPD patients with sarcopenia (COPD‐S). (B) Representative images of hematoxylin and eosin (H&E) staining of lung tissues (upper panel) and gastrocnemius muscles (lower panel). Scale bars, 100 μm. (C) Quantification of serum TNF‐α concentrations in the indicated mouse groups by ELISA. (D) Relative mRNA expression of *Tnf‐α* in the gastrocnemius muscles of the indicated mouse groups, as determined by qRT‐PCR. (E) Representative Western blot analysis of TNF‐α protein expression in the gastrocnemius muscles of the indicated mouse groups. GAPDH was used as a loading control. All data presented as mean ± SD. ***p < 0.001, ****p < 0.0001.

### 3.2 High‐Throughput Sequencing Reveals Activation of the p53 Signaling Pathway in Myoblasts Treated With TNF‐α

To explore the downstream molecular mechanisms by which TNF‐α regulates myoblast function, we performed high‐throughput RNA sequencing on C2C12 cells treated with TNF‐α. Quality control analysis of the sequencing data showed a uniform TPM distribution across samples, and Principal Component Analysis (PCA) demonstrated a clear separation between the control and TNF‐α‐treated groups with high intra‐group repeatability, confirming the reliability of the model and the high quality of the data (Figure S1). On this basis, volcano plot and Venn diagram analyzes revealed that TNF‐α treatment significantly altered the gene expression profile compared to the control (CTRL) group (Figure 2A,B). To identify the key biological pathways involved, we conducted KEGG enrichment analysis, which clearly showed that the p53 signaling pathway was significantly activated among the differentially expressed genes (Figure 2C). This finding was further corroborated by GSEA, which showed a significant positive enrichment of the p53 signaling pathway in the TNF‐α‐treated group (Figure 2D). These data suggest that the p53 signaling pathway may be a critical downstream pathway mediating the biological effects of TNF‐α.

**FIGURE 2:**
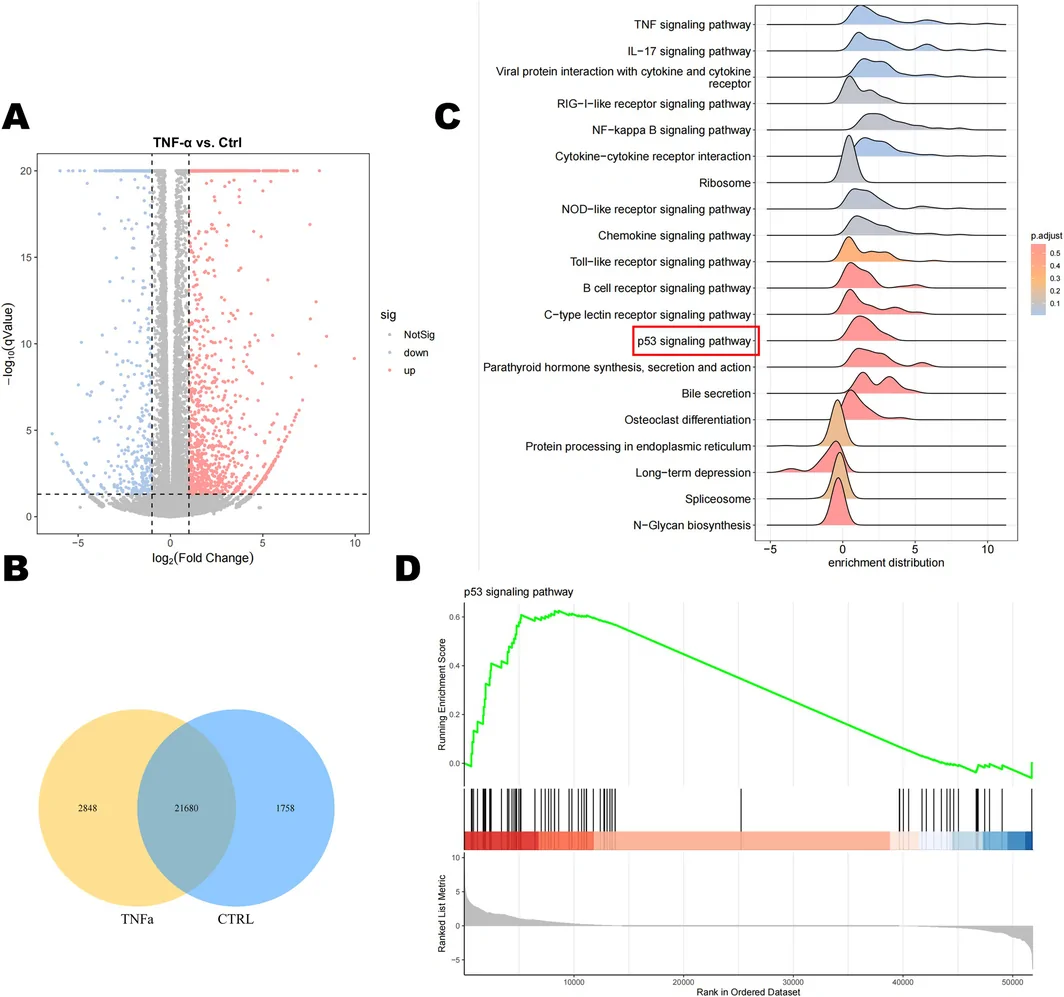
High‐throughput sequencing reveals activation of the p53 signaling pathway in myoblasts treated with TNF‐α. (A) Volcano plot showing DEGs in C2C12 cells treated with TNF‐α (10 ng/mL, 72 h) versus control (CTRL) cells. Red dots indicate significantly upregulated genes, and blue dots indicate significantly downregulated genes. (B) Venn diagram illustrating the overlap of expressed genes between the two groups. (C) KEGG pathway enrichment analysis of the DEGs. The p53 signaling pathway is highlighted with a red box. (D) GSEA plot for the p53 signaling pathway, showing its significant positive enrichment in the TNF‐α‐treated group.

### 3.3 Prodh2 Is a Key Mediator of TNF‐α‐Induced Mitochondrial Stress and Cellular Dysfunction

To validate whether Prodh2, a key molecule identified from sequencing, is regulated by TNF‐α, we examined its expression levels. Both qPCR and Western blot results showed that the mRNA and protein levels of Prodh2 were significantly elevated in C2C12 cells following TNF‐α treatment (Figure 3A,B). A dose‐gradient study indicated that Prodh2 protein expression and cellular damage increased in a dose‐dependent manner with TNF‐α (Figure 3C,D).

**FIGURE 3:**
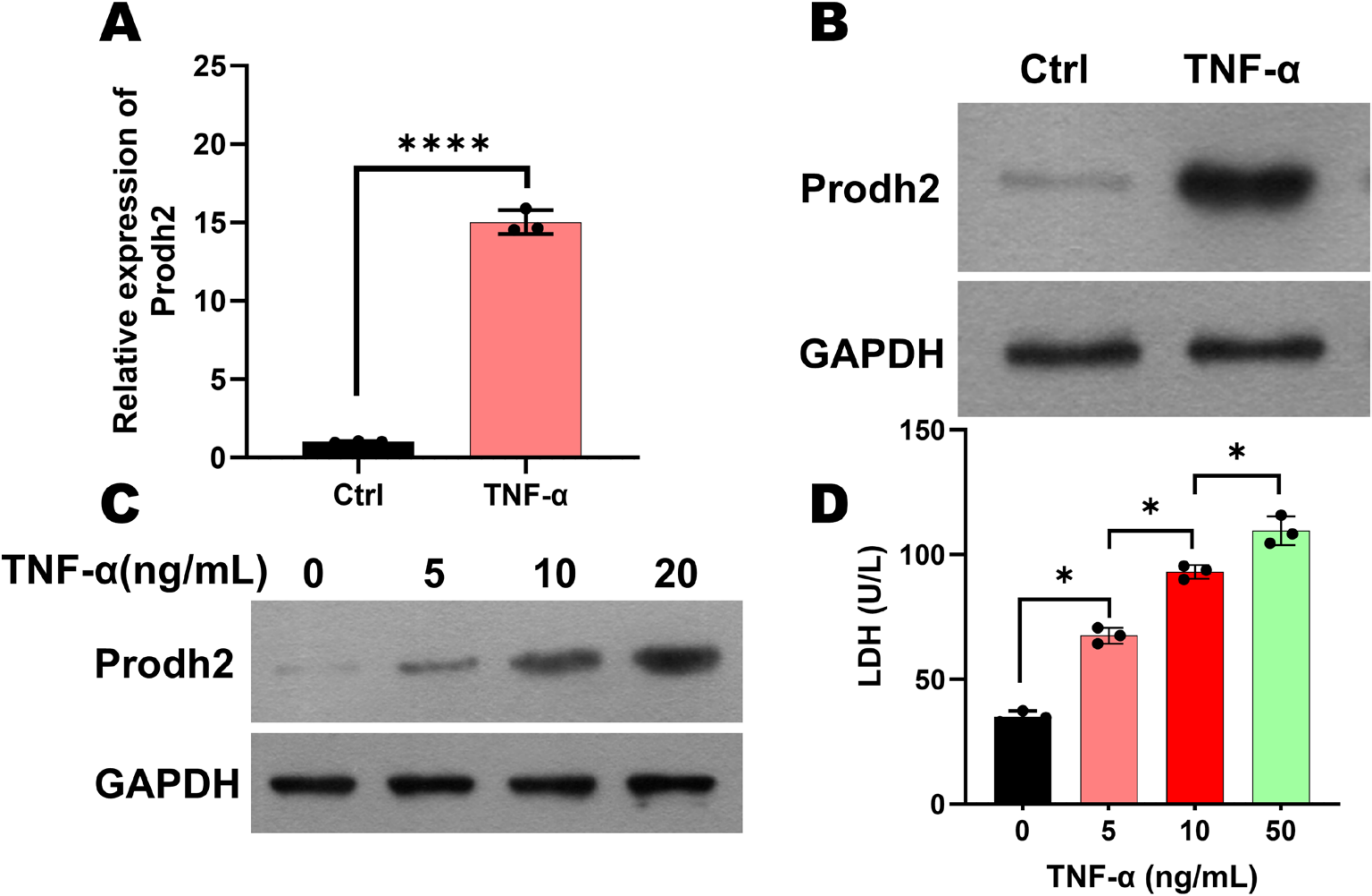
TNF‐α upregulates Prodh2 expression and induces cytotoxicity in C2C12 cells. (A, B) C2C12 cells were treated with TNF‐α (10 ng/mL) for 72 h. The mRNA and protein expression levels of *Prodh2* were determined by qRT‐PCR (A) and Western blot (B), respectively. (C) Western blot analysis of Prodh2 protein expression in C2C12 cells treated with increasing concentrations of TNF‐α (0, 5, 10, 20 ng/mL) for 72 h. (D) Cytotoxicity of C2C12 cells treated with increasing concentrations of TNF‐α (0, 5, 10, 50 ng/mL) for 72 h, as measured by an LDH release assay. All data presented as mean ± SD. *p < 0.05, ****p < 0.0001.

To investigate the necessity of Prodh2 in mediating TNF‐α's effects, we knocked it down using siRNA technology. While knockdown of Prodh2 alone had no significant effect on basal cellular phenotypes, it robustly blocked the detrimental effects induced by subsequent TNF‐α treatment. Specifically, Prodh2 knockdown significantly prevented TNF‐α‐induced mitochondrial ROS production (MitoSox Red) and mitochondrial membrane potential dissipation (JC‐1). Furthermore, it completely abrogated the TNF‐α‐mediated phosphorylation of the signaling molecules STING, IRF3, and p53 (Figure 4). Functionally, the absence of Prodh2 reversed the TNF‐α‐induced increase in cytoplasmic mtDNA (Figure 5A), cytotoxicity (LDH) (Figure 5B), apoptosis (TUNEL, Cleaved Caspase‐3) (Figure 5E,G), and proliferation inhibition (EdU) (Figure 5F). It also prevented the upregulation of muscle atrophy genes Atrogin‐1 and MuRF1 (Figure 5C,D). Morphologically, TNF‐α treatment led to a significant reduction in myotube length, a key feature of muscle atrophy, which was completely rescued by Prodh2 knockdown (Figure 5H). These results establish Prodh2 as a core molecule mediating the effects of TNF‐α.

**FIGURE 4:**
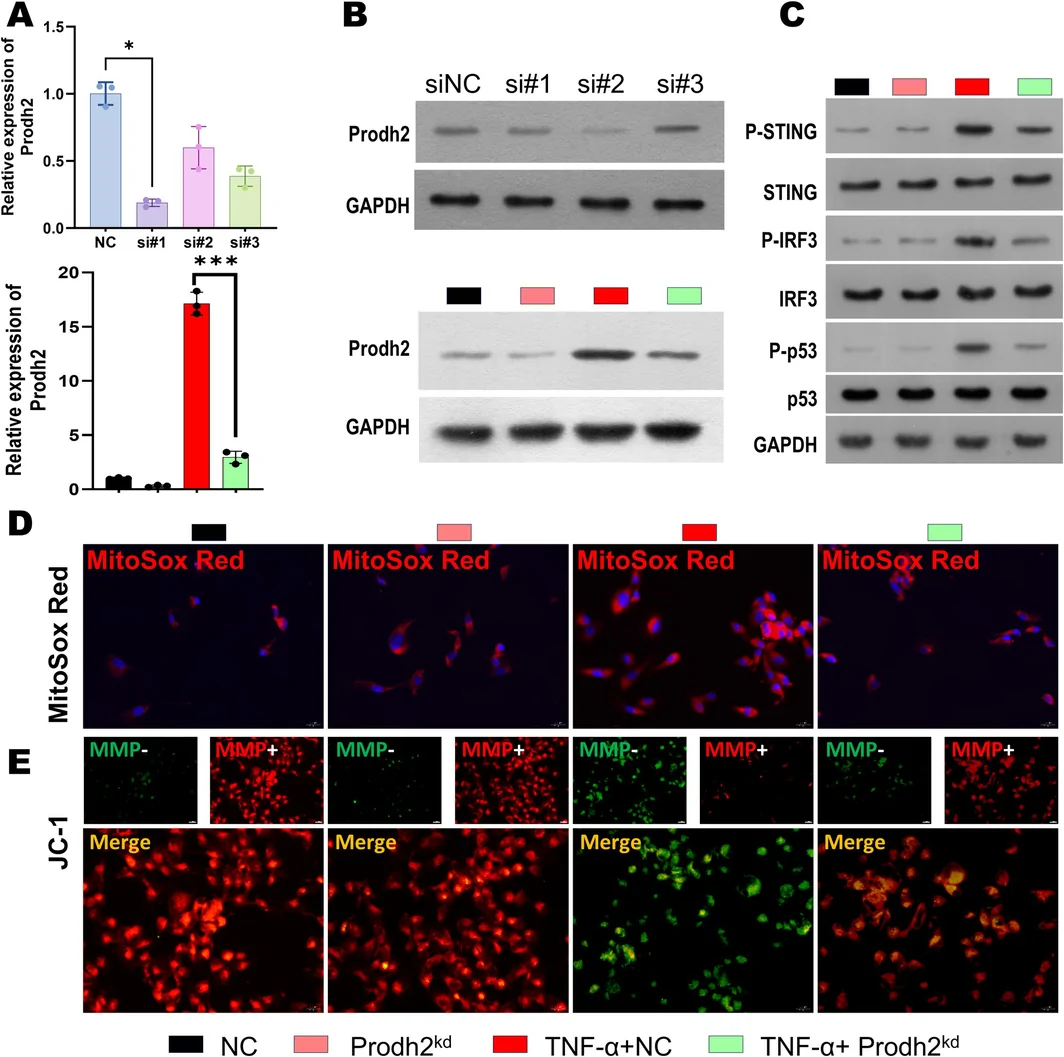
Knockdown of Prodh2 blocks TNF‐α‐induced mitochondrial stress and signaling pathway activation. (A) Knockdown efficiency of three siRNAs targeting *Prodh2* (si#1, si#2, si#3) relative to a negative control siRNA (NC), as determined by qRT‐PCR. (B) Validation of siRNA knockdown efficiency by Western blot. (C) Western blot analysis of the protein levels of p53, STING, IRF3, and their phosphorylated forms in the indicated treatment groups. (D) Mitochondrial ROS levels in the indicated groups, detected by MitoSox Red staining (red fluorescence). Nuclei were counterstained with DAPI (blue). Scale bar, 20 μm (E) Mitochondrial membrane potential in the indicated groups, assessed by JC‐1 staining. Red fluorescence indicates high membrane potential, while green fluorescence indicates low membrane potential. The lower qRT‐PCR and Western blot panels show Prodh2 expression levels in the respective functional experiment groups. siNC, negative control siRNA; Prodh2kd, Prodh2 knockdown Scale bar, 20 μm. All data presented as mean ± SD. *p < 0.05, ***p < 0.001.

**FIGURE 5:**
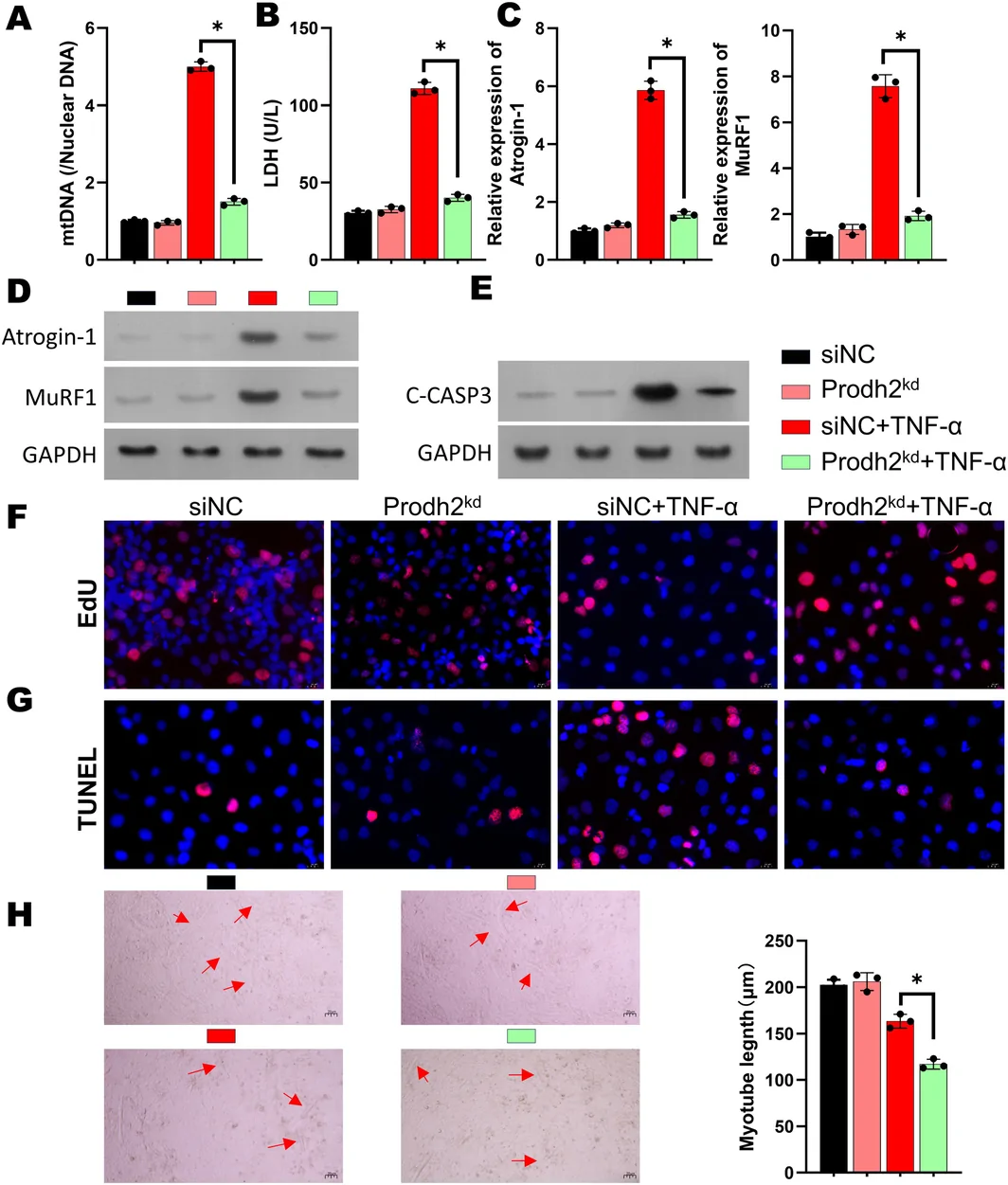
Prodh2 is necessary for TNF‐α‐induced dysfunction in C2C12 cells. C2C12 cells transfected with siNC or si‐Prodh2 were treated with TNF‐α (10 ng/mL), and the following indicators were assessed: (A) Relative levels of cytoplasmic mtDNA, quantified by qRT‐PCR. (B) Cytotoxicity, measured by LDH release assay. (C) mRNA levels of muscle atrophy‐related genes *Atrogin‐1* and *MuRF1*, determined by qRT‐PCR. (D) Protein levels of Atrogin‐1 and MuRF1, determined by Western blot. (E) Protein level of the apoptosis marker Cleaved Caspase‐3 (C‐CASP3), determined by Western blot. (F) Cell proliferation, assessed by EdU incorporation assay (red fluorescence). Scale bar, 20 μm. (G) Apoptosis, assessed by TUNEL assay (red fluorescence). In (F, G), nuclei were counterstained with DAPI (blue). Scale bar, 20 μm. (H) Representative bright‐field images of C2C12 myotubes. Data of myotubes length are expressed as mean length in micrometers of ten myotubes measured per field. Red arrows indicate representative myotubes. Scale bar, 20 μm. All data presented as mean ± SD. *p < 0.05.

### 3.4 TNF‐α Upregulates Prodh2 in a p53‐Dependent Manner and Drives mtROS‐Mediated STING Pathway Activation

To dissect the complete signaling cascade, we performed a series of inhibitor and rescue experiments. The results showed that the transcriptional upregulation of Prodh2 by TNF‐α is p53‐dependent, as this induction was completely abrogated by the p53 inhibitor, Pifithrin‐α (Figure 6A). Functionally, the inhibition of p53, or the scavenging of mitochondrial ROS with MitoTEMPO, was sufficient to rescue cells from TNF‐α‐induced mitochondrial stress (mtROS production and membrane potential loss), proliferation arrest, apoptosis, and myotube atrophy (Figure 6B–E). Further experiments demonstrated that TNF‐α‐induced phosphorylation of both STING and its substrate IRF3 was completely abrogated by either p53 inhibition (Pifα) or mitochondrial ROS scavenging (MitoTEMPO) (Figure 7E). Crucially, Prodh2 overexpression was sufficient to reinstate STING and IRF3 activation in p53‐inhibited cells, causally linking the metabolic enzyme Prodh2 to innate immune signaling. This pattern of STING activation perfectly mirrored the functional outcomes, including the expression of atrophy markers (Figure 7A,B), inhibition of proliferation (Figure 7C), induction of apoptosis (Figure 7D), and myotube atrophy (Figure 7F). These data collectively uncover a linear signaling cascade wherein TNF‐α activates Prodh2 expression via p53, which in turn triggers mitochondrial ROS production, ultimately leading to the activation of the STING pathway.

**FIGURE 6:**
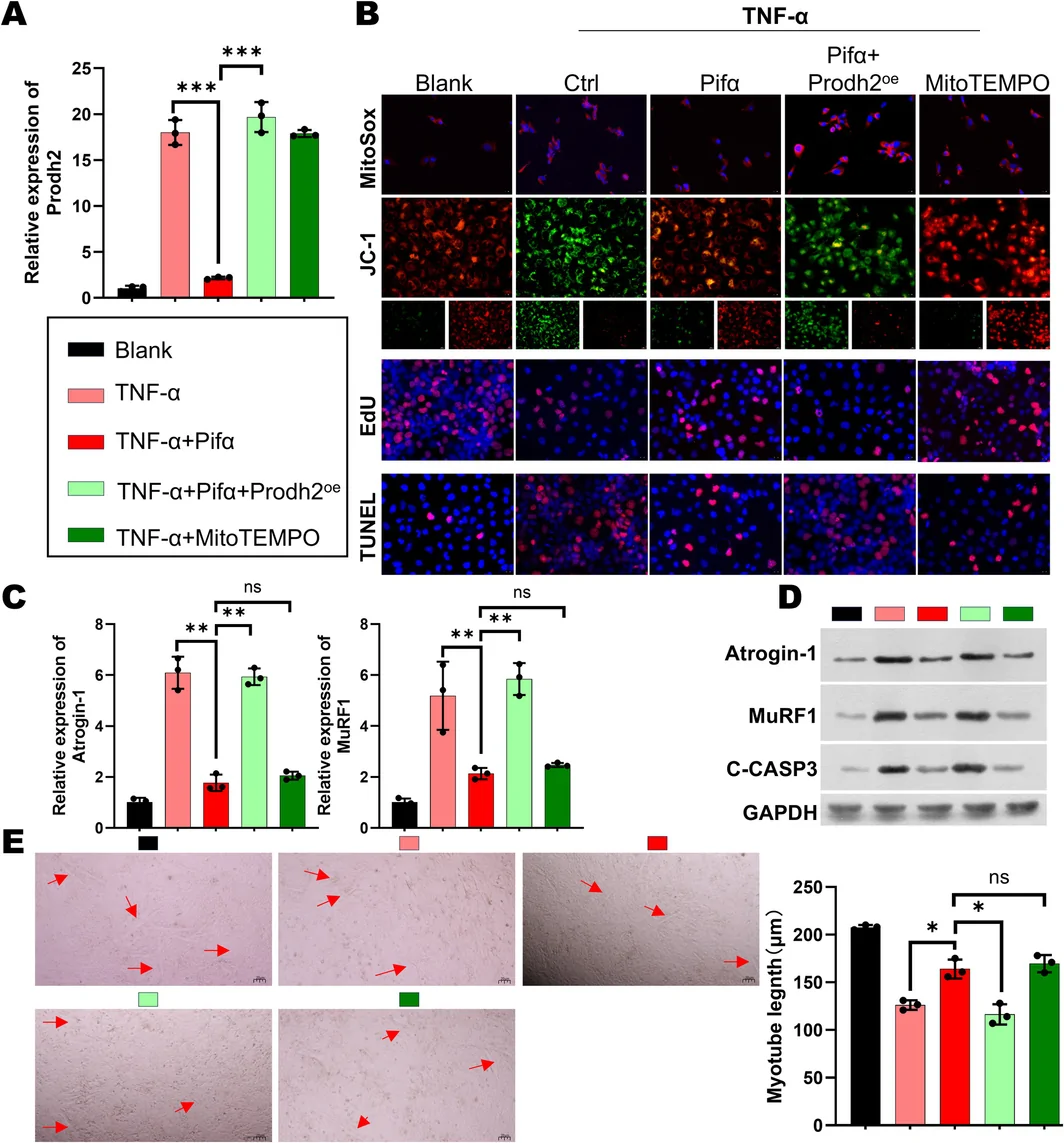
TNF‐α induces cellular dysfunction via the p53‐Prodh2‐mtROS signaling axis. C2C12 cells were treated with TNF‐α (10 ng/mL) in combination with a p53 inhibitor (Pifithrin‐α, Pifα), a Prodh2 overexpression plasmid (Prodh2oe), or a mitochondrial ROS scavenger (MitoTEMPO). (A) Relative mRNA expression of *Prodh2* in the indicated groups, as determined by qRT‐PCR. (B) Representative fluorescence images showing mitochondrial ROS (MitoSox), mitochondrial membrane potential (JC‐1), cell proliferation (EdU), and apoptosis (TUNEL). Scale bar, 20 μm. (C) Relative mRNA expression of *Atrogin‐1* and *MuRF1*, as determined by qRT‐PCR. (D) Western blot analysis of the protein levels of Atrogin‐1, MuRF1, and Cleaved Caspase‐3 (C‐CASP3). (E) Representative bright‐field images of C2C12 myotubes. Data of myotubes length are expressed as mean length in micrometers of ten myotubes measured per field. Red arrows indicate representative myotubes. Scale bar, 20 μm. All data presented as mean ± SD. *p < 0.05, **p < 0.01, ***p < 0.001.

**FIGURE 7:**
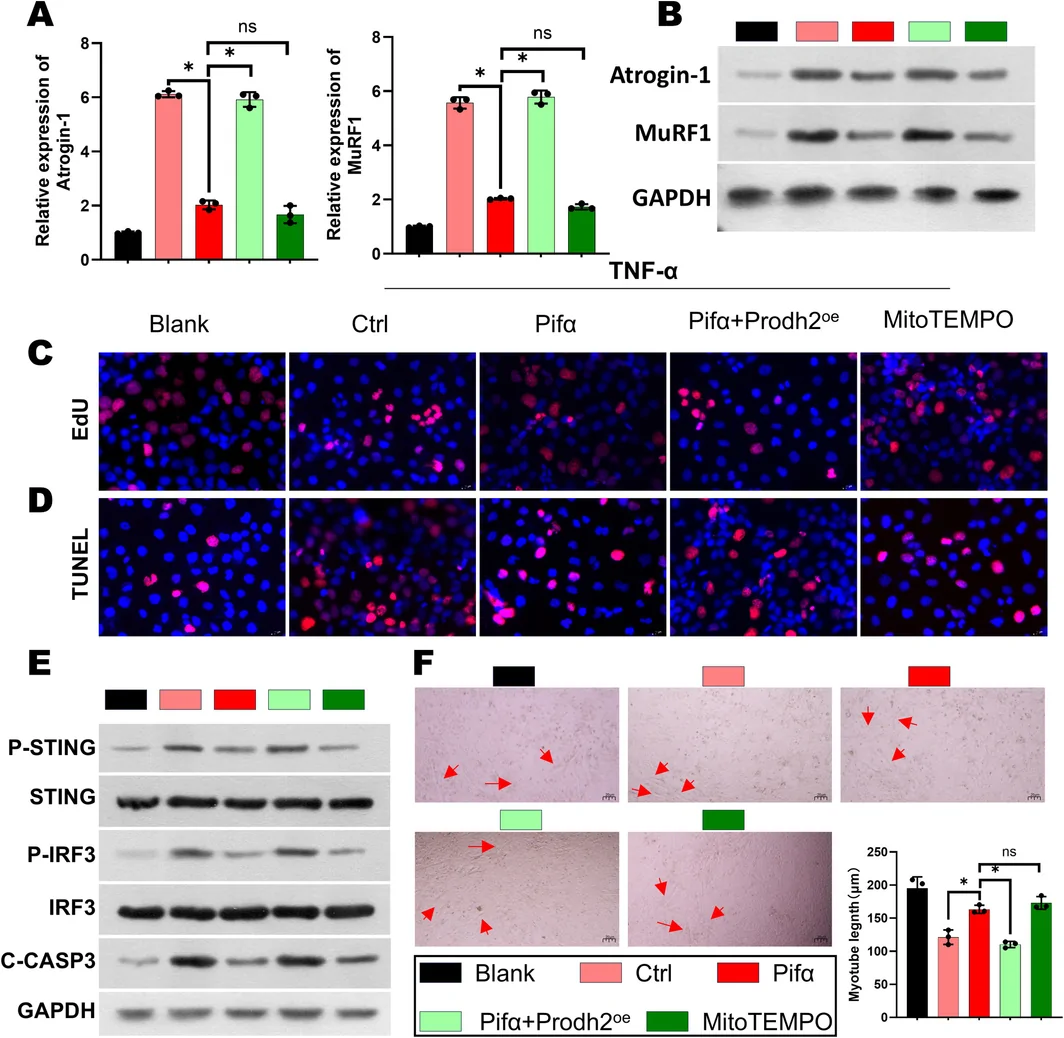
The p53‐Prodh2‐mtROS axis drives downstream STING pathway activation and cellular dysfunction. (A) Relative mRNA expression of *Atrogin‐1* and *MuRF1*, as determined by qRT‐PCR. (B) Western blot analysis of the protein levels of Atrogin‐1 and MuRF1. (C, D) Representative fluorescence images showing cell proliferation (EdU) and apoptosis (TUNEL). Scale bar, 20 μm. (E) Western blot analysis of the protein levels of STING, IRF3, their phosphorylated forms, and Cleaved Caspase‐3 (C‐CASP3). (F) Representative bright‐field images of C2C12 myotubes. Data of myotubes length are expressed as mean length in micrometers of ten myotubes measured per field. Red arrows indicate representative myotubes. Scale bar, 20 μm. All data presented as mean ± SD. *p < 0.05.

### 3.5 Targeted Knockdown of Prodh2 Ameliorates the Muscle Phenotype in a Model of COPD With Sarcopenia

To validate the in vivo efficacy of Prodh2 as a therapeutic target, we performed an AAV‐shRNA intervention in our mouse model of COPD with sarcopenia. Compared to the model control group (nTNF‐α), targeted knockdown of Prodh2 (nTNF‐α + Prodh2kd) significantly improved the grip strength of the mice (Figure 8A) and increased the cross‐sectional area of gastrocnemius muscle fibers (Figure 8D,E). Notably, immunohistochemistry (IHC) analysis revealed the specific spatial distribution of Prodh2 in vivo. In the Se‐Copd model, the robustly upregulated Prodh2 positive signal (brown DAB staining) was diffusely and heavily distributed throughout the cytoplasm of large, polygonal cross‐sections, which are morphological hallmarks of mature contractile myofibers. This histological evidence indicates a universal activation of the Prodh2 pathway in mature muscle tissues under chronic inflammatory stress. Mechanistically, Prodh2 knockdown significantly reduced the expression of atrophy‐related proteins Atrogin‐1 and MuRF1 in muscle tissue (Figure 8C) and effectively inhibited the phosphorylation of p53, IRF3, and STING (Figure 8F). Importantly, knocking down Prodh2 did not affect the high levels of serum TNF‐α in the model mice (Figure 8B). These in vivo results demonstrate that targeted inhibition of Prodh2 effectively ameliorates the COPD‐related sarcopenic phenotype.

**FIGURE 8:**
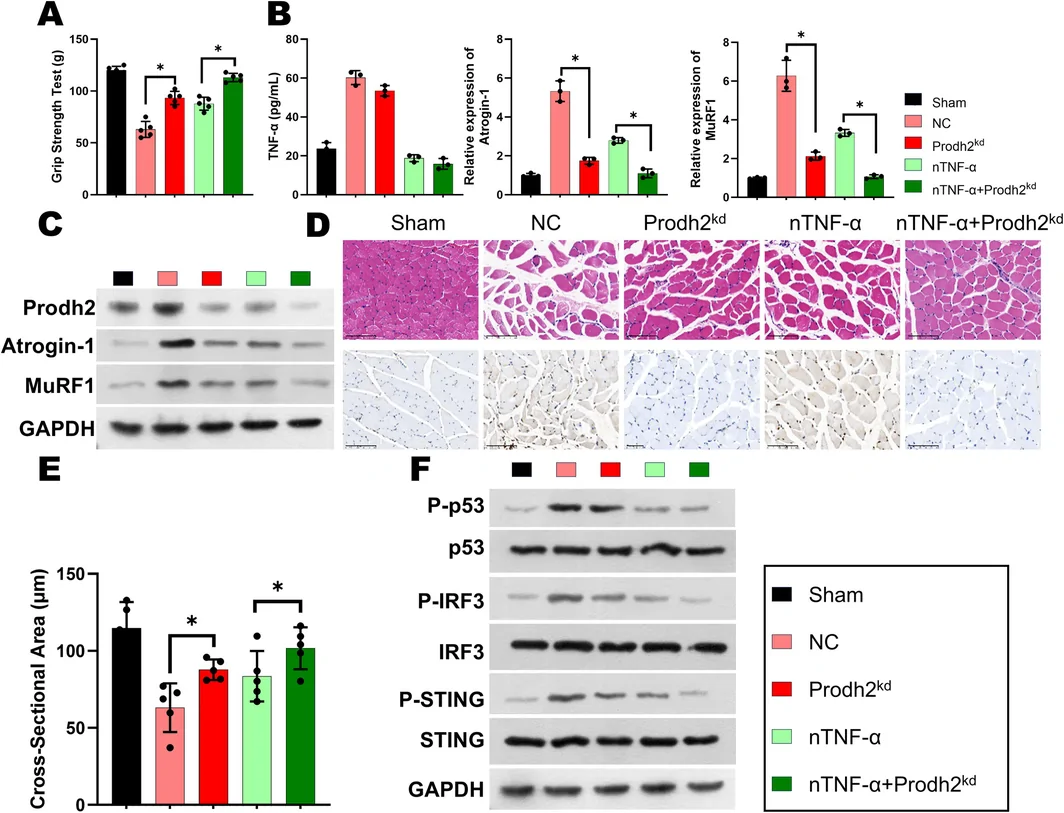
Targeted knockdown of Prodh2 ameliorates the muscle phenotype in a model of COPD with sarcopenia. (A) Grip strength test results for the indicated mouse groups. (B) Serum TNF‐α concentrations determined by ELISA. The right panel shows mRNA expression levels of *Atrogin‐1* and *MuRF1*. (C) Western blot analysis of Prodh2, Atrogin‐1, and MuRF1 protein levels in the gastrocnemius muscles. (D) Representative images of H&E staining (upper panel) and immunohistochemistry (lower panel) of gastrocnemius muscles. Scale bar, 100 μm. (E) Quantification of the cross‐sectional area (CSA) of gastrocnemius muscle fibers. (F) Western blot analysis of p53, IRF3, STING, and their phosphorylated forms in the gastrocnemius muscles. Sham, control group; NC, model + AAV‐shNC group; Prodh2kd, model + AAV‐shProdh2 group; nTNF‐α, model group; nTNF‐α + Prodh2kd, model + AAV‐shProdh2 group. Data are presented as the mean ± SD. *p < 0.05.

## 4 Discussion

This study systematically elucidates the role of Prodh2‐mediated mitochondrial stress in TNF‐α‐induced myoblast dysfunction. We first established the critical role of TNF‐α in COPD‐related sarcopenia through analyzes of clinical samples and an animal model. Subsequently, high‐throughput sequencing led us to identify the p53 signaling pathway and its downstream mitochondrial metabolic enzyme, Prodh2, as core targets of our investigation. Mechanistic studies demonstrated that TNF‐α upregulates Prodh2 in a p53‐dependent manner. The overexpression of Prodh2 drives the production of mitochondrial ROS, leading to mtDNA release and the eventual activation of the cGAS/STING pathway, which triggers myoblast apoptosis and proliferation inhibition. Finally, targeted knockdown of Prodh2 effectively ameliorated the sarcopenic phenotype in our animal model. Collectively, these findings uncover a complete signaling pathway linking chronic inflammation to myoblast dysfunction and suggest that Prodh2 may be a potential target for intervention.

Our baseline data confirm that COPD alone induces skeletal muscle atrophy. This process is primarily driven by chronic systemic inflammation, particularly elevated TNF‐α (Byun et al. 2017; Liao et al. 2026). However, clinical sarcopenia in advanced COPD is multifactorial. Exertional dyspnea frequently forces patients into profound physical inactivity or bed rest (Byun et al. 2017; Matera et al. 2025). Therefore, we utilized the composite “Se‐Copd” model (smoke exposure combined with hindlimb unloading). This “two‐hit” approach better recapitulates the synergistic effects of inflammation and disuse observed in severe clinical cases. Furthermore, classical disuse atrophy relies heavily on mechanical unloading and altered protein degradation, such as the STAT3 or ubiquitin‐proteasome pathways (Wang, Li, et al. 2024; Ji et al. 2024). In contrast, our study reveals a distinct metabolic‐immune axis explicitly driven by the COPD inflammatory milieu. We demonstrate that TNF‐α directly upregulates Prodh2. This enzymatic shift triggers mtROS overproduction and subsequent mtDNA leakage. Consequently, cytosolic mtDNA acts as a danger signal to activate the cGAS/STING innate immune pathway. Similar aberrant mtDNA‐cGAS/STING activation also drives muscle wasting in other severe systemic diseases, such as cirrhosis (Fan et al. 2025). By identifying Prodh2 as the upstream trigger of this immune cascade, our findings bridge the gap between chronic inflammation and innate immunity. This provides a novel mechanistic framework for sarcopenia in advanced COPD.

Proline dehydrogenase 2 (Prodh2) is a mitochondrial enzyme of growing interest in various pathophysiological processes. The metabolic activity of Prodh2 has been shown to enhance CAR‐T cell function in tumor immunology and to promote bone metastasis in breast cancer (Gong, Li, et al. 2025; Ye et al. 2022; Zeng et al. 2022). Additionally, loss‐of‐function mutations in the Prodh2 gene form the molecular basis of hydroxyprolinemia, a benign inherited metabolic disorder (Staufner et al. 2016). However, the function and regulatory mechanisms of Prodh2 in skeletal muscle physiology, particularly in sarcopenia, have not been previously reported. This study reveals a critical role for Prodh2 in the regulation of cellular oxidative stress. The enzymatic reaction of Prodh2 is a source of mitochondrial reactive oxygen species (ROS) (Chalecka et al. 2025), participating in cellular redox homeostasis under physiological conditions. In the context of chronic inflammation driven by TNF‐α, however, Prodh2 expression is aberrantly upregulated. Its sustained catalytic activity is repurposed into a pathological driver, leading to excessive ROS production, sustained mitochondrial oxidative stress, and ultimately, mitochondrial DNA damage and apoptosis. This function of a metabolic enzyme in regulating ROS production and cell fate under stress is evolutionarily conserved, with similar roles reported in plant stress responses (Senthil‐Kumar and Mysore 2012; Bauduin et al. 2022). We traced the aberrant upregulation of Prodh2 to the upstream p53 signaling pathway, providing a direct molecular link between an inflammatory signal and a specific mitochondrial stress. Our results are consistent with literature supporting Prodh2 as a transcriptional target of p53 (Chalecka et al. 2025). Although a previous study reported weak responsiveness of Prodh2 to p53 under acute genotoxic stress in cancer cell lines (Raimondi et al. 2013), such regulation is likely highly dependent on cell type and the nature of the stimulus. In our model, p53 activation by the chronic inflammatory cytokine TNF‐α in myoblasts robustly drives Prodh2 expression. Therefore, our study not only uncovers the pathogenic role of Prodh2 in sarcopenia for the first time but also elucidates the molecular mechanism by which it is precisely regulated by the p53 signaling pathway in a specific inflammatory context.

This study directly links metabolic dysregulation to innate immune activation. Due to its prokaryotic origin, mitochondrial DNA (mtDNA) that leaks into the cytosol acts as a critical damage‐associated molecular pattern (DAMP), which is recognized by the DNA sensor cGAS to activate the STING pathway (Newman and Shadel 2023; Aloraini 2025; Xia et al. 2025). This process triggers a Type I interferon response and “sterile inflammation,” established as a core pathological mechanism in numerous noninfectious diseases, including renal fibrosis, atherosclerosis, and cerebral ischemia–reperfusion injury (Chung et al. 2019; Ueda et al. 2023; Li et al. 2024). The aberrant activation of this pathway is particularly critical in aging and related degenerative conditions, where it serves as a key driver of age‐related chronic inflammation (inflammaging) and is closely associated with cellular senescence and damage in various tissues (Akbari et al. 2021; Jiménez‐Loygorri et al. 2024; Wang, Qi, et al. 2024). Recently, the role of the mtDNA‐cGAS/STING axis in muscle‐wasting diseases has become increasingly evident. In models of COPD‐associated skeletal muscle senescence and cirrhosis‐associated sarcopenia, both elevated cytosolic DNA and STING pathway activation have been shown to contribute to disease progression (Fan et al. 2025; Gong, Shen, et al. 2025). However, the intrinsic upstream mechanisms that directly activate this pathway within myoblasts have remained unclear. The core contribution of our study is the first identification and validation of its precise upstream initiator: a ROS‐dependent mtDNA release process mediated by the specific metabolic enzyme Prodh2. Therefore, our work provides the first complete molecular answer to the critical scientific question of how metabolic dysregulation initiates innate immunity to drive sarcopenia.

This study identifies p53 as a key upstream regulator linking the TNF‐α inflammatory signal to Prodh2‐mediated mitochondrial stress, a finding that highlights a more complex role for p53 beyond its canonical tumor suppressor function. As a central hub of cellular stress, the role of p53 in regulating cellular metabolism and redox homeostasis is increasingly recognized. It has been demonstrated that p53 can not only translocate directly to mitochondria to induce apoptosis through interactions with proteins like Bax, but also control the release of mtDNA by regulating mitochondrial outer membrane permeabilization (Vanpouille‐Box and Galluzzi 2023; Preston et al. 2023). These noncanonical, transcription‐independent functions are highly consistent with the mitochondrial dysfunction and apoptotic phenotypes observed in our model, placing p53 at the center of mitochondrial stress. Our research uncovers a pro‐damage role for p53 in sarcopenia, which is in line with its “maladaptive” function in various noncancerous pathologies. For instance, in models of neurodegenerative diseases, skin aging, and ischemia–reperfusion injury, p53 activation by chronic stress signals, rather than acute DNA damage, similarly drives cellular senescence, apoptosis, and tissue dysfunction (Nan et al. 2025; Wang et al. 2023; Batiha et al. 2023; Zhang et al. 2024). Therefore, our findings do not contradict the established roles of p53 but rather reveal a context‐dependent pathogenic function in the specific pathophysiology of COPD‐related sarcopenia. A critical link in our mechanistic chain is the activation of p53 by TNF‐α, a connection well‐supported by numerous studies. It has been explicitly reported that TNF‐α can directly induce the phosphorylation and activation of p53 in various cell types and mediate cellular senescence through pathways including NF‐κB and p53/p21 (Guo et al. 2025; Aslam et al. 2024). Notably, the transcriptional regulation of *Prodh2* by p53 appears to be highly context‐dependent. While some literature supports *Prodh2* as a p53 target (Raimondi et al. 2013), a comprehensive study by Raimondi et al. found that *Prodh2* was, at best, a very weak p53 target in several cancer cell lines under acute genotoxic stress. The robust p53‐mediated induction of Prodh2 observed in our study may reflect this critical difference in biological context: chronic inflammatory stress (via TNF‐α) in muscle progenitor cells versus acute genotoxic stress in cancer cells. It is plausible that the “inflammation‐p53” axis in myoblasts utilizes distinct transcriptional co‐factors or epigenetic modifications that render the *Prodh2* promoter highly responsive. Thus, the contribution of our study is not merely linking p53 to mitochondrial apoptosis, but more importantly, establishing a complete connection to a specific metabolic effector, Prodh2, for the first time in the field of sarcopenia. This provides a novel mechanistic evidence chain explaining how chronic inflammation, through the classic hub of p53, can precisely regulate mitochondrial metabolic stress to ultimately drive muscle wasting.

Regarding cell‐type specificity, our in vivo IHC data demonstrate that Prodh2 is universally overexpressed in mature myofibers, extending our in vitro findings in myoblasts. We acknowledge that the AAV vector used lacks strict cell‐type specificity as a limitation. Therefore, the observed phenotypic improvements likely result from preserving the universally affected mature myofibers, rather than solely rescuing progenitor cells. Future studies employing cell‐specific Cre‐LoxP systems will further dissect these cell‐autonomous contributions. Additionally, while a systemic wasting phenotype in our study was well established through severe whole‐body grip strength decline and typical gastrocnemius atrophy, the lack of histological data from other representative muscles, such as the diaphragm, is a limitation to be addressed in future investigations.

In conclusion, our study unveils a novel molecular pathway that links chronic inflammation to COPD‐related sarcopenia. We demonstrate that TNF‐α, in a p53‐dependent manner, upregulates the expression of the mitochondrial metabolic enzyme Prodh2. The overexpression of Prodh2 drives the production of mitochondrial ROS, which in turn activates the cGAS/STING innate immune pathway via mtDNA release, ultimately leading to myoblast dysfunction (Graphical Abstract). Our findings not only provide a new “metabolic‐immune crosstalk” perspective for understanding the pathogenesis of sarcopenia but, more importantly, identify Prodh2 as a critical upstream node and a potential therapeutic target for intervention in COPD‐associated muscle wasting.

## Author Contributions

Jingjing Gong and Dalu Cheng conceived and designed the study. Penghong Chen and Jiandong Li collected samples and patient information. Gang Chen and Zhitao Shangguan performed experiments, analyzed the data, and wrote the manuscript. Qiong Lin and Jiandong Li confirm the authenticity of all the raw data. All authors have read and approved the final manuscript.

## Funding

This work was supported by Joint Funds for the innovation of Science and Technology, Fujian province (2021Y9132, 2023Y9152).

## Ethics Statement

The collection and use of human clinical samples were conducted in accordance with the principles of the Declaration of Helsinki. This part of the study was approved by the Institutional Review Board of Fujian Medical University Union Hospital (Approval No: 2023KJCX026). Written informed consent was obtained from all participants prior to their inclusion in the study. All animal experimental protocols were reviewed and approved by the Institutional Animal Care and Use Committee (IACUC) of Fujian Medical University (Approval No: IACUC FJMU 2024‐Y‐1872). All animal procedures were performed in compliance with the Guide for the Care and Use of Laboratory Animals.

## Conflicts of Interest

The authors declare no conflicts of interest.

## Supporting information


**Figure S1:** Quality control of RNA‐sequencing data, related to Figure 2. (A) Violin plot showing the log2‐transformed TPM (transcripts per million) distribution of gene expression levels for each replicate sample (*n* = 3) in the control (CTRL) and TNF‐α‐treated (TNFa) groups. (B) Principal Component Analysis (PCA) plot based on all expressed genes, illustrating the distribution of samples from the two groups along Principal Component 1 (PC1) and Principal Component 2 (PC2).

## Data Availability

The authors declare that the data supporting the findings of this study are available within the paper.
